# Factors Influencing the Healthcare Workers’ Willingness to Receive the COVID-19 Booster Dose in Tuscany (Italy)

**DOI:** 10.3390/vaccines11121751

**Published:** 2023-11-24

**Authors:** Giovanni Guarducci, Giovanna Mereu, Davide Golinelli, Giacomo Galletti, Fabrizio Gemmi, Alessandra Cartocci, Nora Holczer, Luca Bacci, Alessandro Sergi, Gabriele Messina, Valerio Mari, Nicola Nante

**Affiliations:** 1 Post Graduate School of Public Health, University of Siena, 53100 Siena, Italy; davide.golinelli@auslromagna.it (D.G.); gabriele.messina@unisi.it (G.M.); nicola.nante@unisi.it (N.N.); 2 Department of Technical Health Professions, Central Tuscany Local Health Authority, 50121 Florence, Italy; giovanna.mereu@uslcentro.toscana.it; 3 Department of Molecular and Developmental Medicine, University of Siena, 53100 Siena, Italy; 4 Quality and Equity Unit, Regional Health Agency of Tuscany, 50141 Florence, Italy; giacomo.galletti@ars.toscana.it (G.G.); fabrizio.gemmi@ars.toscana.it (F.G.); 5 Department of Medical Biotechnologies, University of Siena, 53100 Siena, Italy; alessandra.cartocci@dbm.unisi.it; 6 General Direction, Central Tuscany Local Health Authority, 50121 Florence, Italyvalerio.mari@uslcentro.toscana.it (V.M.); 7 Web Communication and Promotion, Central Tuscany Local Health Authority, 50121 Florence, Italy; ellebacci@gmail.com; 8 Healthcare Management, Central Tuscany Local Health Authority, 50121 Florence, Italy; alessandro.sergi@uslcentro.toscana.it

**Keywords:** COVID-19, vaccine hesitancy, COVID-19 vaccine, booster dose, trust in vaccine, healthcare workers

## Abstract

Background: The World Health Organization has defined vaccine hesitancy as behavior influenced by several factors, including trust in the vaccine itself or its provider or the perceived need for vaccination. The aim of this study was to investigate the factors influencing the willingness to receive the COVID-19 vaccine among the employees and healthcare professionals of the Central Tuscany Local Health Authority (CT-LHA) in Italy. Methods: From July to October 2022, a cross-sectional study was conducted. An online questionnaire was administered to 7000 employees of the CT-LHA. The questionnaire analyzed the factors that influenced receiving the booster dose of the COVID-19 vaccine. The sample was stratified by gender, age, type of occupation (healthcare or non-healthcare workers), and seniority. Incomplete questionnaires were excluded. A chi-squared test was performed through STATA. The significance level was set at 95%. Results: Of the questionnaires administered, 1885 (26.9%) questionnaires were eligible for the study. In the previous vaccination campaign, the healthcare workers (HCWs) considered the vaccine used by CT-LHA as safe, in contrast to non-healthcare workers (N-HCWs), who considered it less secure (*p* < 0.05). The HCWs showed a higher propensity for vaccine safety to receive the booster dose than N-HCWs. N-HCWs appeared to be less affected by an updated booster dose than HCWs (*p* < 0.05). Conclusions: The factors studied appear to influence HCWs differently from N-HCWs. Both HCWs and N-HCWs would choose an upgraded mRNA vaccine for the booster dose.

## 1. Introduction

Vaccination stands as a cornerstone of global health, playing a pivotal role in public health initiatives [1,2]. Its significance lies in the unmatched ability to avert countless fatalities by instilling immunity against several infectious diseases and alleviating their burden on individuals and society [3,4]. However, despite its undeniable benefits, vaccine hesitancy has emerged as a critical concern flagged by the World Health Organization (WHO) among the top ten global health threats. This intricate phenomenon refers to the delay or outright refusal of vaccines, even when vaccination services are readily available [5,6,7]. While not a novel issue, vaccine hesitancy has gained unprecedented visibility due to the dissemination of anti-vaccination misinformation on social media platforms, further exacerbated by the ongoing SARS-CoV-2 pandemic [5]. The pandemic’s context has witnessed the rapid emergency authorization and approval of numerous COVID-19 vaccines, raising concerns about their safety, potential adverse effects, and long-term impacts due to the lack of longitudinal data [8,9]. Consequently, apprehension and hesitancy toward these vaccines have arisen, prompting the need for healthcare systems to address vaccine hesitancy through evidence-based interventions and to devise COVID-19 vaccine strategies that effectively tackle emerging mutations of the virus [10]. These efforts are vital to ensure the success of vaccination campaigns, leading to the attainment of herd immunity and the containment of infection spread within communities [11]. Vaccine hesitancy can arise from various reasons, encompassing apprehensions about vaccine safety and efficacy, distrust in healthcare systems or pharmaceutical entities, the propagation of misinformation, religious, or philosophical beliefs, and the fear of potential side effects [12,13,14,15,16]. Among certain employee cohorts, such as healthcare workers, vaccine hesitancy has garnered significant attention during the COVID-19 pandemic. Some sectors, including healthcare, have implemented mandatory vaccination mandates in a bid to create a safe working environment. However, resistance from a subgroup of employees hesitant to receive primary and booster vaccine doses has been encountered [17,18]. Addressing vaccine hesitancy among workers necessitates a multifaceted approach [19]. Accurate and easily accessible information on vaccines, coupled with the resolution of concerns and dispelling of myths, is paramount [20]. Employers, as influential stakeholders, can play a pivotal role in creating a supportive and educational environment to positively influence vaccine hesitancy, as reported in the scientific literature [21,22]. Establishing trust and fostering partnerships among employers, healthcare workers, and non-healthcare workers becomes vital in combating vaccine hesitancy within workplace settings [23,24]. It is pertinent to acknowledge that vaccine hesitancy is a multifarious issue, with individual reasons for hesitancy varying widely [7,25].

The present study aims to investigate vaccine hesitancy among employees, encompassing both healthcare and non-healthcare professionals, within the Center Tuscany Local Health Authority in Italy, specifically focusing on the booster dose for COVID-19. By delving into this crucial topic, this study seeks to contribute valuable insights that may aid in addressing vaccine hesitancy and formulating targeted strategies for enhancing vaccination acceptance rates among workforces.

## 2. Materials and Methods

### 2.1. Study Design and Setting

A cross-sectional study (see the STROBE checklist in Supplementary Material S1) was conducted on the employees of the Center Tuscany Local Health Authority (CT-LHA). The CT-LHA has an area of 5000 square kilometers and provides care for approximately 1,500,000 people. It has over 14,000 employees, 13 hospitals, 220 territorial facilities, 8 district zones, and 7 health societies [26].

From 26 July to 15 October 2022, an anonymous and voluntary questionnaire was administered to 7000 employees out of the approximately 14,000 employees of the CT-LHA who had an open-ended or fixed-term contract. The questionnaire, validated by the Regional Health Agency of Tuscany [27], aimed to analyze both the factors influencing receiving the booster dose against COVID-19 and the aspects of the COVID-19 vaccination CT-LHA campaign.

### 2.2. Questionnaire

The questionnaire (Supplementary Material S2) was sent to the institutional e-mail of each participant. They were informed that information would be collected anonymously, treated confidentially, and stored securely for as long as necessary for the data analysis and dissemination. Furthermore, all personal data retrieved were strictly for the purpose of the present analysis, and individual participants cannot be identified based on the material presented. Participation was voluntary, and the questionnaire of those who decided to take part was collected anonymously without, therefore, causing any plausible harm or stigma to the participants or non-participants.

The questionnaire was composed of 14 questions divided into 4 survey sections:

(1) Individual characteristics (gender, age, type of occupation, and seniority);
(2) Vaccination history and aspects of the booster dose of COVID-19 vaccine (previous COVID-19 vaccination history, reasons for choosing the booster dose linked to the employee, and risk of contagion);
(3) Assessments of COVID-19 vaccine (efficacy and safety of the vaccine used for previous COVID-19 vaccinations, motivation for choice to vaccinate, linked to the vaccine and choice of vaccine type available for the booster dose);
(4) Communication, organizational, and access aspects (an evaluation of the anti-COVID-19 vaccination campaign, recognition of competence for technical-scientific aspects, recognition of competence for organizational aspects, and preference for information tools).

### 2.3. Analysis

Our study analyzed the first three survey sections (individual characteristics, vaccination history, and aspects of the booster dose of the COVID-19 vaccine and the assessments of the COVID-19 vaccine).

The individual characteristics associated with sections two and three were stratified as follows:

- Gender: male (M), female (F), and not declared (N.D.);
- Age: from 20 to 35 years, from 36 to 55 years, from 56 to 75 years, and not declared (N.D.);
- Type of profession: healthcare worker, non-healthcare worker, and not declared (N.D.);
- Seniority: from 1 to 5 years, from 6 to 15 years, over 15 years, and not declared (N.D.).

Missing data analysis was performed, and the missing were completely random. Incomplete questionnaires were excluded from the study. Further details and analyses were reported in the Supplementary Materials S3.

Qualitative variables were summarized by absolute frequencies and percentages. A chi-squared test or Fisher exact test was carried out to evaluate the association between individual characteristics and questionnaire items. Post hoc analysis, based on multiple Fisher exact tests and false discovery rate (FDR) correction, was performed when the global chi-squared test was significant. The analyses were carried out with STATA software SE/14.0 (StataCorp LLC, College Station, TX, USA) and R version 4.0.1. A level of 95% (*p* < 0.05) was considered statistically significant.

## 3. Results

Of the 7000 questionnaires administered, 2010 were returned (28.7%), and applying the exclusion criteria (incomplete questionnaires) resulted in 1885 questionnaires being eligible for the study (26.9% of the questionnaires administered and 93.8% of those returned). Table 1 shows the characteristics of the study sample. Most of the enrolled employees were female (73.74%), and only 2.07% did not declare their gender. The most represented age group was between 36 and 55 years old (53.79%), followed by between 56 and 75 years old (40.21%) and between 20 to 35 years old (5.15%). More than two-thirds of the sample were healthcare workers (69.44%), and only 1.54 did not declare their occupation. Two-thirds of the sample (66.63%) had more than 15 years of seniority, followed by between 6 and 15 years of seniority, and then by less than 5 years, and those who did not declare were 1.17%.

Table 2 shows the doses of the anti-COVID-19 vaccine doses received divided by gender, age group, type of occupation, and seniority. Most of the sample had received three doses of COVID-19 vaccine. According to gender, males and females showed a similar propensity to receive three doses. All age groups had received almost completely two or three doses. For the third dose, just a 3% difference was observed between the younger and the older cohorts. No significant difference was observed between types of occupation in vaccination against COVID-19. Seniority seems associated with the number of doses received; however, no significant differences were observed when comparing the various groups.

Table 3 shows the factors that influenced employees in choosing the booster dose, stratified by gender, age group, type of occupation, and seniority. Those who had between 36 and 55 showed little fear of infection than the older (39.8% vs. 48.9% Very Much/Much) and were more influenced by obligation (44.2% vs. 37.9% Very Much/Much) (*p* < 0.05). Females appeared to be much more affected by mandatory vaccination than males.

Table 4 shows the influencing factors related to the risk of infection in choosing the booster dose, divided by gender, age group, type of occupation, and seniority. According to the risk of infection, all age groups would choose vaccination in the same proportion in the three options. Although the younger cohorts’ answers were statistically different from the elders, and in particular, the younger cohort considered they were more probable to be infected (35.1% vs. 29.9% for Neither Much nor Little, 30.9% vs. 34.3% Very Little/Little) and infecting others (37.1% vs. 29.0% Neither Much nor Little, 33.0% vs. 44.3% Very Little/Little). Seniority follows the same trend of age. Employees with less seniority were considered statistically more probable to be infected (45.3% vs. 36.2% Very Much/Much) and to infect others (36.8% vs. 30.9% Very Much/Much) than the older ones (*p* < 0.05).

Table 5 shows the evaluation of the vaccine used in the anti-COVID-19 campaign divided by gender, age group, type of occupation, and seniority. The males assessed the vaccine used by the CT-LHA as both very effective and safe, in contrast to the females, who found it to be moderately effective and low in safety (*p* < 0.05): 50.7% vs. 37.1% and 53.5 vs. 40.8% for the Very High/High answer, respectively. The younger employees considered it safer than the middle-aged group; the latter considered it less secure than the older employees (*p* < 0.05). The HCWs considered the vaccine used by the CT-LHA as safe, in contrast to N-HCWs, who considered it less secure (*p* < 0.05). The employees with more than 15 years of seniority considered that the vaccine used was less safe than those with less seniority (*p* < 0.05).

Table 6 shows the vaccine-related influencing factors leading to undergoing the booster dose, stratified by gender, age group, type of occupation, and seniority. In receiving the booster dose, the females appeared less influenced by safety, efficacy, and vaccine update than the males (about a 10% difference for the Very High/High option), *p* < 0.05. The HCWs showed a higher propensity to receive the booster dose for safety than N-HCWs and those who had not declared their occupation. N-HCWs appeared to be less affected by an updated booster dose than HCWs (*p* < 0.05).

Table 7 shows the preferred vaccine for the booster dose (the mRNA, viral vector, mRNA updated for the Omicron variant, and indifferent) by gender, age group, type of occupation, and seniority. Most of the sample chose the mRNA-updated vaccine (about 60% in each group). No substantial differences were shown between groups, except for the N-HCWs, who chose the “Indifferent” option about 10% more than HCWs (*p* < 0.05). 

## 4. Discussion

Vaccine-hesitant individuals comprise a heterogeneous group with varying degrees of indecision regarding specific vaccines or vaccination in general; they may accept all, some, or none of the available vaccines [28]. Our study aimed to identify the potential factors influencing vaccine hesitancy for the booster dose of the anti-COVID-19 vaccine cohort among employees of an Italian Local Health Authority. The CT-LHA of Tuscany is responsible for the care needs of approximately 1,500,000 citizens (more than 40% of the overall Tuscan population) [26,29] and was impacted by the pandemic, especially during the second wave [30]. A questionnaire was designed to ascertain the reasons for their choice. In a pandemic scenario, protecting employees is of paramount importance, particularly for healthcare workers. The recent SARS-CoV-2 pandemic underscored the susceptibility of healthcare workers to airborne pathogens, particularly in the absence of suitable personal protective equipment or vaccine prophylaxis [31,32]. Our study revealed that no gender differences in receiving vaccination for COVID-19 were present. This contradicts other studies that suggested females were less likely to opt for vaccination [33,34]. This may be because the CT-LHA has done a good job in offering fair and gender-neutral access to the possibility of vaccination. Unlike other studies [33], our results did not show an increase in the likelihood of vaccination with advancing age. Despite an early vaccination of healthcare workers during the pandemic or a higher degree of hesitancy towards the vaccine among non-healthcare workers [33,35,36], no discrepancies between occupational types in the anti-COVID-19 vaccination cohort were found in our study. This result underlines, again, how CT-LHA managed to involve all types of employees in the vaccination program. For older employees, fear of infection played a crucial role in influencing their decisions. Indeed, self-protection from COVID-19 and the consequent fear of contracting the disease were influential factors in the decision to receive the booster dose, as seen in another study [37]. In our study, this appeared for people over 56 rather than for those between 36 and 55. Mandatory vaccination policies may not always be the most effective approach to reducing population hesitancy [38,39]. However, in our study, they seemed to significantly influence the decision to receive the booster dose by females and those ages between 36 and 55 compared to older employees. Balancing the duty to work against fear of infection and potential transmission to others was a crucial consideration for frontline employees during the pandemic [40,41,42]. Frequently, this insecurity and fear of infection or transmission were exacerbated by inadequate information, limited access to personal protective equipment, and subpar crisis management at the workplace [42,43]. In our study, we found that a fear of contracting the virus and a fear of transmitting it was greatly influenced by not having received the booster dose among all age subgroups compared to those who had not declared their age.

Perceptions of the safety and efficacy of vaccination are not always uniform [44,45,46,47]. The male gender was generally positively associated with anti-COVID-19 vaccination [32,48,49]; in our study, the males (in contrast to females) rated the COVID-19 vaccine they received as very effective and safe. Similar perceptions were observed among healthcare workers, who considered the vaccine they received as very safe. When deciding to receive the booster dose, the male gender was highly influenced by the perceived efficacy and safety, while females were mildly influenced by safety and moderately by efficacy. One of the most common reasons for vaccine hesitancy is concern over safety [50,51]. Healthcare workers, compared with non-healthcare workers, underwent the booster dose because they evaluated it as safe, which seemed to positively influence their decision. It is likely that non-healthcare workers, due to a lack of medical knowledge and understanding of COVID-19 vaccines, were less concerned about vaccine safety [36]. Although no differences appeared with efficacy and safety concerns among older versus younger people, one study found that the latter may be influenced by increased exposure to social media [51].

The reasons for COVID-19 vaccination hesitancy among healthcare workers are complex and diverse. These concerns align with those observed for other vaccines and in the general population. However, the rapid development of COVID-19 vaccines using innovative technologies introduced unique elements of hesitancy during the pandemic. When addressing vaccine hesitancy, it is crucial to recognize the array of concerns healthcare providers may have [52]. Indeed, achieving herd immunity, along with other public health measures, is key to both pandemic preparedness and public health responses [53].

### Limitations

Despite the significant findings of this study, it is essential to recognize its limitations. Firstly, this research was conducted within a single setting: an Italian local health authority. Although there are several studies in the literature, this is the first study to analyze the attitudes of employees of an LHA in the Tuscany region. Therefore, the findings may not be directly applicable to other contexts or regions, potentially limiting the generalizability of our results. The specific characteristics of this population, including cultural attitudes toward healthcare and immunization, might have influenced the results. Thus, additional studies in diverse settings are necessary to provide a more comprehensive understanding of the factors influencing vaccine hesitancy. Finally, our study describes the behaviors of the analyzed subjects without using a multivariate analysis, as it was not the aim of our study to identify the main factors influencing them.

Secondly, our study lacks information about the individuals who did not complete the questionnaire. Their reasons for non-completion could range from a lack of time or interest to a potential hesitancy or resistance towards the vaccine. This absence of data creates a potential response bias, as those who chose not to participate may have different attitudes and behaviors towards vaccination than those who participated, which could have influenced our findings.

Lastly, the study design itself poses a limitation. As a cross-sectional study, it only provides a snapshot of attitudes and behaviors at a particular point in time, making it difficult to infer causality or track changes over time. While this design is efficient and effective for assessing the prevalence of vaccine hesitancy and identifying related factors, it does not allow for the examination of how these factors may evolve and interact over time, nor does it identify cause-and-effect relationships. Further longitudinal research is needed to address these limitations to provide a more nuanced understanding of vaccine hesitancy. While our findings contribute valuable insights into the factors influencing vaccine hesitancy for the COVID-19 booster dose, limitations must be considered when interpreting the results. Future research addressing these limitations can provide further depth and breadth to our understanding of this important issue.

## 5. Conclusions

Ensuring appropriate vaccination of at-risk individuals, including healthcare workers, is the optimal strategy to prevent SARS-CoV-2 infection and its potential severe complications. As healthcare workers could become a source of infection if unvaccinated, it is essential to combat and mitigate the effects of vaccination hesitancy. This requires investigation and intervention of the main determinants of this phenomenon, such as a misperception of the disease or distrust of vaccines. At the same time, it is equally critical to examine the psychological and cognitive mechanisms that underpin vaccination-related behaviors, including the influence of social regulations. While changing people’s beliefs or feelings is a challenging goal, particularly in a controversial topic like vaccination, it is possible to facilitate behavioral changes, especially among the hesitant. In the realm of vaccination, the most impactful strategies are those that directly target behaviors.

The findings of our study suggest the importance of continuous education and information dissemination for improving employee adherence to vaccination programs, perhaps initiating this process during the new recruitment procedure. By promoting a culture of safety, we can equip all employees, specifically healthcare workers, with the necessary knowledge to implement safety strategies for themselves, patients, and users. By focusing on newly recruited young individuals, we can foster an awareness of the risks they face due to their healthcare context, irrespective of their youth or health. Further, mandatory training modules should be included in local health authority training plans on vaccinations, wherein the safety and side effects of the vaccine are clearly explained, and the concept of community immunity is thoroughly addressed. This can potentially counteract those who wish to benefit from herd immunity by letting others get vaccinated. Allowing employees to compare their facility’s vaccination adherence data with other settings, where better adherence to vaccination is noted, could serve as a vaccination incentive. Measuring adherence can impact the operational and decision-making processes in each setting, allowing not only for widespread knowledge dissemination about the risk of illness due to non-vaccination but also for the adoption of improvement actions to overcome critical issues in a shared manner.

Lastly, we propose that future efforts should focus on enhancing the quality of communication regarding the disease under investigation and the efficacy of vaccines. This is necessary to mitigate the negative effects of insufficient or distorted information. Furthermore, training healthcare and non-healthcare workers in risk knowledge and the corresponding ability to avoid it are key components of the behavior change process.

## Figures and Tables

**Table 1:** Characteristics of the sample.

Sample Characteristics	Number	%
Gender		
Male (M)	456	24.19
Female (F)	1390	73.74
Not Declared (N.D.)	39	2.07
Total	1885	100.00
Age		
From 20 to 35 years	97	5.15
From 36 to 55 years	1014	53.79
From 56 to 75 years	758	40.21
N.D.	16	0.85
Total	1885	100.00
Occupation		
Healthcare workers (HWCs)	1309	69.44
Non-Healthcare workers (N-HWCs)	547	29.02
Not Declared (N.D.)	29	1.54
Total	1885	100.00
Seniority		
From 1 to 5 years	106	5.62
From 6 to 15 years	501	26.58
Over 15 years	1256	66.63
Not Declared (N.D.)	22	1.17
Total	1885	100.00

**Table 2:** Number of anti-COVID-19 vaccine doses received by gender, age, occupation, and seniority.

Doses Received—N° (%)					
Answer		None	Only One	Only Two	Three
Total		4	10	49	1822
Gender	F	2 (0.1)	6 (0.4)	38 (2.7)	1344 (96.8)
	M	0 (0.0)	0 (0.0)	11 (2.4)	445 (97.6)
	N.D.	2 (5.1)	4 (10.3)	0 (0.0)	33 (84.6)
*p*		<0.001 ^b,c^			
Age	20–35	0 (0.0)	0 (0.0)	5 (5.2)	92 (94.8)
	36–55	2 (0.1)	9 (0.9)	29 (2.9)	974 (96.1)
	56–75	1 (0.1)	0 (0.0)	15 (2.0)	742 (97.9)
	N.D.	1 (6.3)	1 (6.3)	0 (0.0)	14 (87.4)
*p*		0.002 ^c,d,e,f^			
Occupation	HCW	2 (0.2)	6 (0.5)	37 (2.8)	1264 (96.5)
	N-HCW	1 (0.2)	3 (0.6)	12 (2.4)	531 (96.8)
	N.D.	1 (3.5)	1 (3.5)	0 (0.0)	27 (93.0)
*p*		0.072			
Seniority	1–5	0 (0.0)	0 (0.0)	0 (0.0)	106 (100.0)
	6–15	1 (0.2)	7 (1.4)	17 (3.4)	476 (95.0)
	≥16	2 (0.2)	3 (0.3)	32 (2.5)	1219 (97.0)
	N.D.	1 (4.5)	0 (0.0)	0 (0.0)	21 (95.5)
*p*		0.014			
Fisher exact test was carried out for the global association. ^a^ F was statistically different from M; ^b^ F was statistically different from N.D.; ^c^ M was statistically different from N.D. ^a^ 20–35 was statistically different from 36–55; ^b^ 20–35 was statistically different from 56–75.; ^c^ 20–35 was statistically different from N.D.; ^d^ 36–55 was statistically different from 56–75; ^e^ 36–55 was statistically different from N.D.; ^f^ 56–75 was statistically different from N.D. ^a^ HCW was statistically different from N-HCW; ^b^ HCW was statistically different from N.D.; ^c^ N-HCW was statistically different from N.D. ^a^ 1–5 was statistically different from 6–15; ^b^ 1–5 was statistically different from ≥16; ^c^ 1–5 was statistically different from N.D.; ^d^ 6–15 was statistically different from ≥16; ^e^ 6–15 was statistically different from N.D.; ^f^ ≥16 was statistically different from N.D.					

**Table 3:** Employee-related influencing factors related to choosing the booster dose by type, gender, age, occupation, and seniority.

		Factors Influencing the Choice of the Booster Dose—N° (%)								
		Fear of Infection			Fear of Infecting Others			Obligation		
Answer		Very Much/Much	Neither Much nor Little	Very Little/Little	Very Much/Much	Neither Much nor Little	Very Much/Much	Neither Much nor Little	Very Little/Little	Very Much/Much
Total		817	197	871	833	207	845	778	213	894
Gender	F	595 (42.8)	148 (10.6)	647 (46.5)	609 (43.8)	150 (10.8)	631 (45.4)	604 (43.5)	145 (10.4)	641 (46.1)
	M	208 (45.6)	47 (10.3)	201 (44.1)	211 (46.3)	55 (12.1)	190 (41.7)	165 (36.2)	59 (12.9)	232 (50.9)
	N.D.	14 (35.9)	2 (5.1)	23 (59.0)	13 (33.3)	2 (5.1)	24 (61.5)	9 (23.1)	9 (23.1)	21 (53.8)
*p*		0.391			0.145			0.003 ^a,b^		
Age	20–35	39 (40.2)	7 (7.2)	51 (52.6)	41 (42.3)	12 (12.4)	44 (45.4)	41 (42.3)	10 (10.3)	46 (47.4)
	36–55	404 (39.8)	116 (11.4)	494 (48.7)	427 (42.1)	114 (11.2)	473 (46.6)	448 (44.2)	115 (11.3)	451 (44.5)
	56–75	371 (48.9)	73 (9.6)	314 (41.4)	362 (47.8)	80 (10.6)	316 (41.7)	287 (37.9)	88 (11.6)	383 (50.5)
	N.D.	3 (18.8)	1 (6.3)	12 (75.0)	3 (18.8)	1 (6.3)	12 (75.0)	2 (12.5)	0 (0.0)	14 (87.5)
*p*		0.001 ^d^			0.063			0.006 ^c,d,e,f^		
Occupation	HCW	567 (43.3)	141 (10.8)	601 (45.9)	584 (44.6)	147 (11.2)	578 (44.2)	531 (40.6)	152 (11.6)	626 (47.8)
	N-HCW	240 (43.9)	54 (9.9)	253 (46.3)	241 (44.1)	58 (10.6)	248 (45.3)	237 (43.3)	60 (11.0)	250 (45.7)
	N.D.	10 (34.5)	2 (6.9)	17 (58.6)	8 (27.6)	2 (6.9)	19 (65.5)	10 (34.5)	1 (3.4)	18 (62.1)
*p*		0.698			0.250			0.344		
Seniority	1–5	48 (45.3)	12 (11.3)	46 (43.4)	53 (50.0)	11 (10.4)	42 (39.6)	44 (41.5)	17 (16.0)	45 (42.5)
	6–15	188 (37.5)	56 (11.2)	257 (51.3)	207 (41.3)	64 (12.8)	230 (45.9)	216 (43.1)	55 (11.0)	230 (45.9)
	≥16	572 (45.5)	129 (10.3)	555 (44.2)	564 (44.9)	132 (10.5)	560 (44.6)	512 (40.8)	141 (11.2)	603 (48.0)
	N.D.	9 (40.9)	0 (0.0)	13 (59.1)	9 (40.9)	0 (0.0)	13 (59.1)	6 (27.3)	0 (0.0)	16 (72.7)
*p*		0.043			0.245			0.123		
Chi-squared test was carried out for the global association. ^a^ F was statistically different from M; ^b^ F was statistically different from N.D.; ^c^ M was statistically different from N.D. ^a^ 20–35 was statistically different from 36–55; ^b^ 20–35 was statistically different from 56–75.; ^c^ 20–35 was statistically different from N.D.; ^d^ 36–55 was statistically different from 56–75; ^e^ 36–55 was statistically different from N.D.; ^f^ 56–75 was statistically different from N.D. ^a^ HCW was statistically different from N-HCW; ^b^ HCW was statistically different from N.D.; ^c^ N-HCW was statistically different from N.D. ^a^ 1–5 was statistically different from 6–15; ^b^ 1–5 was statistically different from ≥16.; ^c^ 1–5 was statistically different from N.D.; ^d^ 6–15 was statistically different from ≥16; ^e^ 6–15 was statistically different from N.D.; ^f^ ≥16 was statistically different from N.D.										

**Table 4:** Employee-related influencing factors in choosing the booster dose, related to the risk of infection, by type, gender, age, occupation, and seniority.

		Not Having Received the Booster Dose Considers It Likely—N (%)					
		Being Infected			Infecting Others		
Answer		Very Much/Much	Neither Much nor Little	Very Little/Little	Very Much/Much	Neither Much nor Little	Very Little/Little
Total		675	567	643	584	535	766
Gender	F	490 (35.3)	428 (30.8)	472 (34.0)	427 (30.7)	391 (28.2)	572 (41.2)
	M	172 (37.7)	133 (29.2)	151 (33.1)	147 (32.2)	138 (30.3)	171 (47.5)
	N.D.	13 (33.3)	6 (15.4)	20 (51.3)	10 (25.6)	6 (15.4)	23 (59.0)
*p*		0.120			0.093		
Age	20–35	33 (34.0)	34 (35.1)	30 (30.9)	29 (29.9)	36 (37.1)	32 (33.0)
	36–55	369 (36.4)	306 (30.2)	339 (33.4)	337 (33.2)	294 (29.0)	383 (37.8)
	56–75	271 (35.8)	227 (29.9)	260 (34.3)	218 (28.8)	204 (26.9)	336 (44.3)
	N.D.	2 (12.5)	0 (0.0)	30 (30.9)	0 (0.0)	1 (6.2)	15 (93.8)
*p*		0.001 ^c,e,f^			<0.001 ^c,d,e,f^		
Occupation	HCW	489 (37.4)	393 (30.0)	427 (32.6)	416 (31.8)	381 (29.1)	512 (39.1)
	N-HCW	174 (31.8)	171 (31.3)	202 (36.9)	158 (28.9)	151 (27.6)	238 (43.5)
	N.D.	12 (41.4)	3 (10.3)	14 (48.3)	10 (34.5)	3 (10.3)	16 (55.2)
*p*		0.022			0.084		
Seniority	1–5	48 (45.3)	33 (31.1)	25 (23.6)	39 (36.8)	42 (39.6)	25 (23.6)
	6–15	168 (33.5)	168 (33.5)	165 (32.9)	154 (30.7)	159 (31.7)	188 (37.5)
	≥16	455 (36.2)	361 (28.7)	440 (35.0)	388 (30.9)	330 (26.3)	538 (42.8)
	N.D.	4 (18.2)	5 (22.7)	13 (59.1)	3 (13.6)	4 (18.2)	15 (68.2)
*p*		0.011 ^c^			<0.001 ^a,b,c,e^		
Chi-squared test was carried out for the global association. ^a^ F was statistically different from M; ^b^ F was statistically different from N.D.; ^c^ M was statistically different from N.D. ^a^ 20–35 was statistically different from 36–55; ^b^ 20–35 was statistically different from 56–75.; ^c^ 20–35 was statistically different from N.D.; ^d^ 36–55 was statistically different from 56–75; ^e^ 36–55 was statistically different from N.D.; ^f^ 56–75 was statistically different from N.D. ^a^ HCW was statistically different from N-HCW; ^b^ HCW was statistically different from N.D.; ^c^ N-HCW was statistically different from N.D. ^a^ 1–5 was statistically different from 6–15; ^b^ 1–5 was statistically different from ≥16.; ^c^ 1–5 was statistically different from N.D.; ^d^ 6–15 was statistically different from ≥16; ^e^ 6–15 was statistically different from N.D.; ^f^ ≥16 was statistically different from N.D.							

**Table 5:** Evaluation of the vaccine used in previous anti-COVID-19 vaccination campaign by gender, age, occupation, and seniority.

		How Do You Evaluate the Vaccine Used for Employees?—N° (%)					
		Efficacy			Safety		
Answer		Very High/High	Medium	Very Low/Low	Very High/High	Medium	Very Low/Low
Total		756	710	419	823	685	377
Gender	F	515 (37.1)	556 (40.0)	319 (22.9)	567 (40.8)	519 (37.3)	304 (21.9)
	M	231 (50.7)	138 (30.3)	87 (19.1)	244 (53.5)	151 (33.1)	61 (13.4)
	N.D.	10 (25.6)	16 (41.1)	13 (33.3)	12 (30.8)	15 (38.5)	12 (30.8)
*p*		<0.001 ^a,c^			<0.001 ^a^		
Age	20–35	34 (35.1)	42 (43.3)	21 (21.6)	57 (58.8)	27 (27.8)	13 (13.4)
	36–55	392 (38.7)	365 (36.0)	257 (25.3)	426 (42.0)	349 (34.4)	239 (23.6)
	56–75	324 (42.7)	296 (39.1)	138 (18.2)	426 (56.2)	303 (44.0)	122 (16.1)
	N.D.	6 (37.5)	7 (43.8)	3 (18.8)	7 (43.8)	6 (37.5)	3 (18.8)
*p*		0.023			<0.001 ^a,d^		
Occupation	HCW	541 (41.3)	476 (47.7)	292 (22.3)	624 (47.7)	442 (33.8)	243 (18.6)
	N-HCW	206 (37.7)	221 (34.4)	120 (21.9)	188 (43.4)	231 (42.2)	128 (23.4)
	N.D.	9 (31.0)	13 (37.9)	7 (24.1)	11 (37.9)	12 (41.4)	6 (20.7)
*p*		0.400			<0.001 ^a^		
Seniority	1–5	48 (45.3)	39 (61.3)	19 (17.9)	65 (61.3)	27 (25.5)	14 (13.2)
	6–15	207 (41.3)	183 (46.3)	111 (22.2)	232 (46.3)	170 (33.9)	99 (19.8)
	≥16	494 (39.3)	477 (41.1)	285 (22.7)	516 (41.1)	480 (38.2)	260 (20.7)
	N.D.	7 (31.8)	11 (45.5)	4 (18.2)	10 (45.5)	8 (36.4)	4 (18.2)
*p*		0.712			0.005 ^b^		
Chi-squared test was carried out for the global association. ^a^ F was statistically different from M; ^b^ F was statistically different from N.D.; ^c^ M was statistically different from N.D. ^a^ 20–35 was statistically different from 36–55; ^b^ 20–35 was statistically different from 56–75.; ^c^ 20–35 was statistically different from N.D.; ^d^ 36–55 was statistically different from 56–75; ^e^ 36–55 was statistically different from N.D.; ^f^ 56–75 was statistically different from N.D. ^a^ HCW was statistically different from N-HCW; ^b^ HCW was statistically different from N.D.; ^c^ N-HCW was statistically different from N.D. ^a^ 1–5 was statistically different from 6–15; ^b^ 1–5 was statistically different from ≥16.; ^c^ 1–5 was statistically different from N.D.; ^d^ 6–15 was statistically different from ≥16; ^e^ 6–15 was statistically different from N.D.; ^f^ ≥16 was statistically different from N.D.							

**Table 6:** Vaccine-related influencing factors by gender, age, occupation, and seniority.

		You Would Undergo the Booster Dose Because—N° (%)								
		Effective			Safe			Updated		
Answer		Very High/High	Medium	Very Low/Low	Very High/High	Medium	Very Low/Low	Very High/High	Medium	Very Low/Low
Total		621	565	699	700	567	618	584	495	806
Gender	F	425 (30.6)	437 (31.4)	528 (38.0)	479 (34.5)	429 (30.9)	482 (34.7)	410 (29.5)	384 (27.6)	596 (42.9)
	M	188 (41.2)	119 (26.1)	149 (32.7)	213 (46.7)	128 (28.1)	115 (25.2)	168 (36.8)	102 (22.4)	186 (40.8)
	N.D.	8 (20.5)	9 (23.1)	22 (56.4)	8 (20.5)	10 (25.6)	21 (53.8)	9 (23.1)	9 (23.1)	24 (61.5)
*p*		<0.001 ^a,c^			<0.001 ^a,b,c^			0.002 ^a^		
Age	20–35	32 (33.0)	23 (23.7)	42 (43.3)	40 (41.2)	22 (22.7)	33 (34.0)	40 (41.2)	25 (25.8)	39 (40.2)
	36–55	319 (31.5)	303 (29.9)	392 (38.7)	358 (35.3)	309 (30.5)	347 (34.2)	308 (30.4)	264 (26.0)	442 (43.6)
	56–75	266 (35.1)	238 (31.4)	254 (33.5)	299 (39.4)	233 (30.7)	226 (29.8)	240 (31.7)	203 (26.8)	315 (41.6)
	N.D.	4 (25.0)	1 (6.3)	11 (68.8)	3 (18.8)	3 (18.8)	10 (62.5)	3 (18.8)	3 (18.8)	10 (62.5)
*p*		0.017			0.032			0.708		
Occupation	HCW	444 (33.9)	396 (30.3)	469 (35.8)	531 (40.6)	389 (29.7)	389 (29.7)	420 (32.1)	361 (27.6)	528 (40.3)
	N-HCW	170 (31.1)	163 (29.8)	214 (39.1)	164 (30.0)	172 (31.4)	211 (38.6)	159 (29.1)	127 (23.2)	261 (47.7)
	N.D.	7 (24.1)	6 (20.7)	16 (55.2)	5 (17.2)	6 (20.7)	18 (62.1)	5 (17.2)	7 (24.1)	17 (58.6)
*p*		0.182			<0.001 ^a,b^			0.014 ^a^		
Seniority	1–5	39 (36.8)	30 (28.3)	37 (34.9)	50 (47.2)	27 (25.5)	29 (27.4)	38 (35.8)	30 (28.3)	38 (35.8)
	6–15	179 (35.7)	140 (27.9)	182 (36.3)	191 (38.1)	149 (29.7)	161 (32.1)	160 (31.9)	130 (25.9)	211 (42.1)
	≥16	399 (31.8)	387 (30.8)	470 (37.4)	455 (36.2)	384 (30.6)	417 (33.2)	382 (30.4)	322 (25.6)	542 (43.2)
	N.D.	4 (18.2)	8 (36.4)	10 (45.5)	4 (18.2)	7 (31.8)	11 (50.0)	4 (18.2)	3 (13.6)	15 (68.2)
*p*		0.459			0.153			0.211		
Chi-squared test was carried out for the global association. ^a^ F was statistically different from M; ^b^ F was statistically different from N.D.; ^c^ M was statistically different from N.D. ^a^ 20–35 was statistically different from 36–55; ^b^ 20–35 was statistically different from 56–75.; ^c^ 20–35 was statistically different from N.D.; ^d^ 36–55 was statistically different from 56–75; ^e^ 36–55 was statistically different from N.D.; ^f^ 56–75 was statistically different from N.D. ^a^ HCW was statistically different from N-HCW; ^b^ HCW was statistically different from N.D.; ^c^ N-HCW was statistically different from N.D. ^a^ 1–5 was statistically different from 6–15; ^b^ 1–5 was statistically different from ≥16.; ^c^ 1–5 was statistically different from N.D.; ^d^ 6–15 was statistically different from ≥16; ^e^ 6–15 was statistically different from N.D.; ^f^ ≥16 was statistically different from N.D.										

**Table 7:** Preferred booster vaccine type by gender, age, occupation, and seniority.

Which Vaccine Would You Choose for the Booster Dose—N° (%)					
Answer		mRNA	Viral Vector	mRNA, updated	Indifferent
Total		267	28	1153	437
Gender	F	185 (13.3)	17 (1.2)	871 (62.7)	317 (22.8)
	M	77 (16.9)	11 (2.4)	261 (57.2)	107 (23.5)
	N.D.	9 (23.1)	0 (0.0)	21 (53.8)	13 (33.3)
*p*		0.093			
Age	20–35	19 (19.6)	0 (0.0)	53 (54.6)	25 (25.8)
	36–55	148 (14.6)	14 (1.4)	599 (59.1)	253 (25.0)
	56–75	99 (13.1)	14 (1.8)	495 (65.3)	150 (19.8)
	N.D.	1 (6.3)	0 (0.0)	6 (37.5)	9 (56.3)
*p*		0.005			
Occupation	HCW	197 (15.0)	17 (1.3)	835 (63.8)	260 (19.9)
	N-HCW	66 (12.1)	11 (2.0)	307 (56.1)	163 (29.8)
	N.D.	4 (13.8)	0 (0.0)	11 (37.9)	14 (48.3)
*p*		<0.001 ^a,b^			
Seniority	1–5	13 (12.3)	0 (0.0)	70 (66.0)	23 (21.7)
	6–15	75 (15.0)	7 (1.4)	289 (57.7)	130 (25.9)
	≥16	175 (13.9)	21 (1.7)	785 (62.5)	275 (21.9)
	N.D.	4 (18.2)	0 (0.0)	9 (40.9)	9 (40.9)
*p*		0.219			
Chi-squared test was carried out. ^a^ F was statistically different from M; ^b^ F was statistically different from N.D.; ^c^ M was statistically different from N.D. ^a^ 20–35 was statistically different from 36–55; ^b^ 20–35 was statistically different from 56–75.; ^c^ 20–35 was statistically different from N.D.; ^d^ 36–55 was statistically different from 56–75; ^e^ 36–55 was statistically different from N.D.; ^f^ 56–75 was statistically different from N.D. ^a^ HCW was statistically different from N-HCW; ^b^ HCW was statistically different from N.D.; ^c^ N-HCW was statistically different from N.D. ^a^ 1–5 was statistically different from 6–15; ^b^ 1–5 was statistically different from ≥16.; ^c^ 1–5 was statistically different from N.D.; ^d^ 6–15 was statistically different from ≥16; ^e^ 6–15 was statistically different from N.D.; ^f^ ≥16 was statistically different from N.D.					

## Data Availability

Data are contained within the article and supplementary materials. Other information about data of this study are available on request from the corresponding author.

## Supplementary Materials

The following supporting information can be downloaded at: https://www.mdpi.com/article/10.3390/vaccines11121751/s1, S1: STROBE statement checklist; S2: Translated questionnaire; S3: Missing data analysis.
