# Supplementary material for: Factors Influencing the Healthcare Workers’ Willingness to Receive the COVID-19 Booster Dose in Tuscany (Italy)
Source: Vaccines (Basel). 2023 Nov 24;11(12):1751. doi: 10.3390/vaccines11121751 (PMC10748028; doi:10.3390/vaccines11121751)
Supplement: Supplementary file 1 [file vaccines-11-01751-s001.zip › vaccines-2695397-supplementary.pdf]

Supplementary Material

Table S1. STROBE Statement-Checklist.

	Item No	Recommendation	Page No
**Title and abstract**	1	(*a*) Indicate the study's design with a commonly used term in the title or the abstract	1
		(*b*) Provide in the abstract an informative and balanced summary of what was done and what was found	1
**Introduction**			
Background/rationale	2	Explain the scientific background and rationale for the investigation being reported	1, 2
Objectives	3	State specific objectives, including any prespecified hypotheses	2
**Methods**			
Study design	4	Present key elements of study design early in the paper	2
Setting	5	Describe the setting, locations, and relevant dates, including periods of recruitment, exposure, follow-up, and data collection	2, 3
Participants	6	(*a*) Give the eligibility criteria, and the sources and methods of selection of participants	3
Variables	7	Clearly define all outcomes, exposures, predictors, potential confounders, and effect modifiers. Give diagnostic criteria, if applicable	3
Data sources/ measurement	8*	For each variable of interest, give sources of data and details of methods of assessment (measurement). Describe comparability of assessment methods if there is more than one group	*3*
Bias	9	Describe any efforts to address potential sources of bias	3
Study size	10	Explain how the study size was arrived at	2, 3
Quantitative variables	11	Explain how quantitative variables were handled in the analyses. If applicable, describe which groupings were chosen and why	3
Statistical methods	12	(*a*) Describe all statistical methods, including those used to control for confounding	3
		(*b*) Describe any methods used to examine subgroups and interactions	3
		(*c*) Explain how missing data were addressed	3
		(*d*) If applicable, describe analytical methods taking account of sampling strategy	2, 3
		(*e*) Describe any sensitivity analyses	-
**Results**			
Participants	13*	(a) Report numbers of individuals at each stage of study—e.g., numbers potentially eligible, examined for eligibility, confirmed eligible, included in the study, completing follow-up, and analysed	3
		(b) Give reasons for non-participation at each stage	3

		(c) Consider use of a flow diagram	-
Descriptive data	14*	(a) Give characteristics of study participants (e.g., demographic, clinical, social) and information on exposures and potential confounders	3, 4
		(b) Indicate number of participants with missing data for each variable of interest	-
Outcome data	15*	Report numbers of outcome events or summary measures	5-9
Main results	16	(*a*) Give unadjusted estimates and, if applicable, confounder-adjusted estimates and their precision (e.g., 95% confidence interval). Make clear which confounders were adjusted for and why they were included	5-9
		(*b*) Report category boundaries when continuous variables were categorized	-
		(*c*) If relevant, consider translating estimates of relative risk into absolute risk for a meaningful time period	-
Other analyses	17	Report other analyses done—e.g., analyses of subgroups and interactions, and sensitivity analyses	5-9
**Discussion**			
Key results	18	Summarise key results with reference to study objectives	10, 11
Limitations	19	Discuss limitations of the study, taking into account sources of potential bias or imprecision. Discuss both direction and magnitude of any potential bias	11
Interpretation	20	Give a cautious overall interpretation of results considering objectives, limitations, multiplicity of analyses, results from similar studies, and other relevant evidence	11, 12
Generalisability	21	Discuss the generalisability (external validity) of the study results	10, 11
**Other information**			
Funding	22	Give the source of funding and the role of the funders for the present study and, if applicable, for the original study on which the present article is based	12

*Give information separately for exposed and unexposed groups.

Supplementary material S2. Translated questionnaire.

**Which is your Gender?**			
☐ Male	☐ Female	☐ I prefer not to declare it	
**What age group are you in?**			
☐ From 20 to 35 years	☐ From 36 to 55 years	☐ From 56 to 75 years	☐ I prefer not to declare it
**What kind of employee are you?**			
☐ Healthcare workers	☐ Non-Healthcare workers	☐ I prefer not to declare it	
**How long have you been employed by Central Tuscany Local Health Authority (CT-LHA)?**			
☐ From 1 to 5 years	☐ From 6 to 15 years	☐ Over 15 years	☐ I prefer not to declare it

**How many doses of COVID-19 vaccine did you receive?**			
☐ None	☐ Only one	☐ Only two	☐ Three

**How much do you think the following reasons might influence your decision to undergo the booster dose for COVID-19?**					
**Fear of infection**	☐ Very much	☐ Much	☐ Neither much nor little	☐ Little	☐ Very Little
**Fear of infecting others**	☐ Very much	☐ Much	☐ Neither much nor little	☐ Little	☐ Very Little
**Obligation**	☐ Very much	☐ Much	☐ Neither much nor little	☐ Little	☐ Very Little

**Not having received the booster dose for COVID-19, how likely do you think it is to:**					
**Being infected**	☐ Very much	☐ Much	☐ Neither much nor little	☐ Little	☐ Very Little
**Infecting others**	☐ Very much	☐ Much	☐ Neither much nor little	☐ Little	☐ Very Little

**How do you assess the following aspects of the vaccine used in the COVID-19 campaign for the workers in CT-LHA?**					
**Efficacy**	☐ Very High	☐ High	☐ Medium	☐ Low	☐ Very Low
**Safety**	☐ Very High	☐ High	☐ Medium	☐ Low	☐ Very Low

**Why would you undergo the booster dose of COVID-19 vaccine?**					
**Effective**	☐ Very High	☐ High	☐ Medium	☐ Low	☐ Very Low
**Safe**	☐ Very High	☐ High	☐ Medium	☐ Low	☐ Very Low
**Updated**	☐ Very High	☐ High	☐ Medium	☐ Low	☐ Very Low

**Which type of COVID--19 vaccine would you choose for the booster dose?**			
☐ mRNA	☐ Viral Vector	☐ mRNA updated	☐ Indifferent

**How do you assess the following aspects in the vaccination campaign for employees of CT-LHA?**					
**Quality of communication**	☐ Very High	☐ High	☐ Medium	☐ Low	☐ Very Low
**Ease of booking**	☐ Very High	☐ High	☐ Medium	☐ Low	☐ Very Low
**Ease of access to vaccination hubs**	☐ Very High	☐ High	☐ Medium	☐ Low	☐ Very Low

**From whom would you like to receive information on the technical and scientific aspects of the booster dose of the COVID--19 vaccine?**		
☐ Corporate Task Force	☐ Occupational physician	☐ Ministry of Health Tutorial
☐ General Practitioner	☐ Company Intranet	☐ Other

From whom would you like to receive information regarding the logistic-organizational aspects of the COVID--19 booster dose?		
☐ Medical Direction	☐ Prevention Department	☐ Vaccination physicians
☐ Corporate Task Force	☐ Other	
**What tools would you prefer for disseminating information on the booster dose of the COVID-19 vaccine?**		
☐ Institutional e-mail	☐ WhatsApp	☐ Operating Procedures
☐ Internal circulars	☐ Other	

Supplementary material S3. Missing data analysis.

Analyses were carried out with R version 4.0.1 and the package "finalfit". Chi-squared test was carried out to evaluate an association between missing/non missing data of the questionnaire item and the variable of interest. Missing are present only on 125 out of 2010 observations.

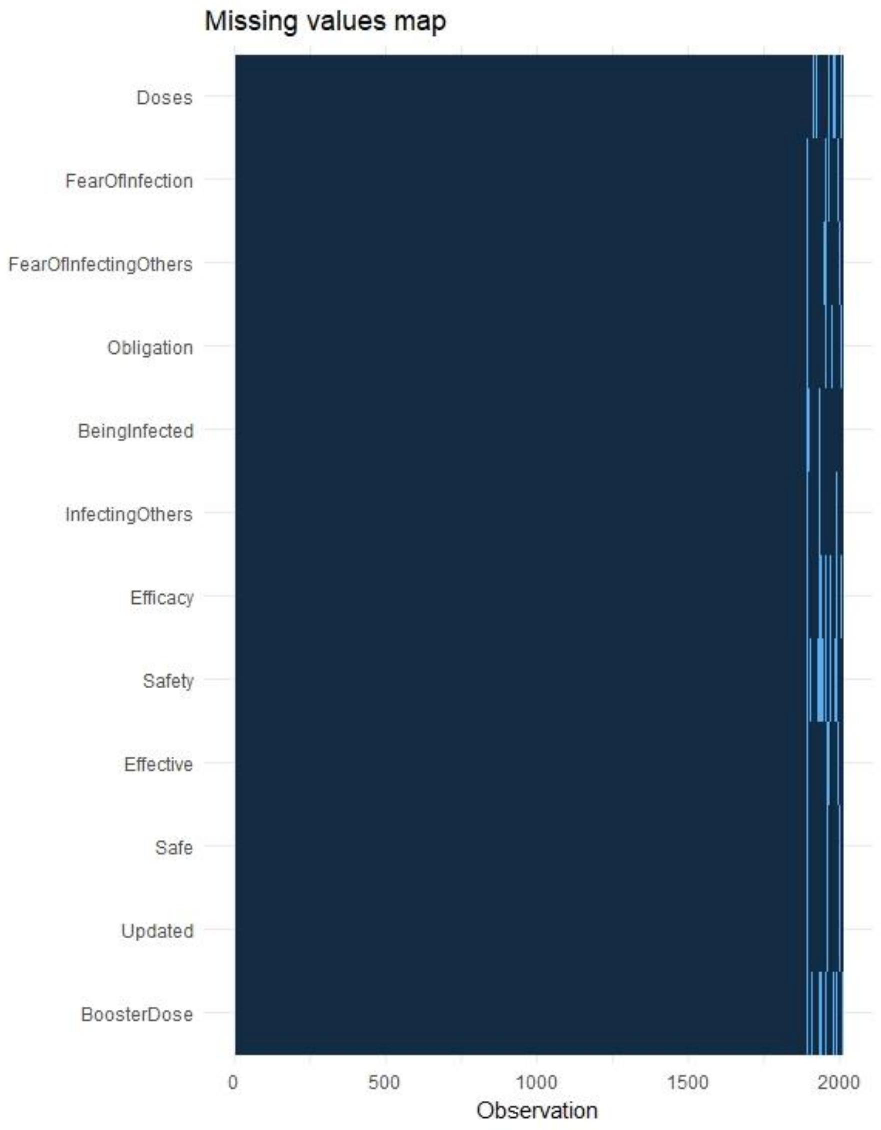

Here, chi-squared test results were reported:

Missing data analysis: Doses		Not missing	Missing	p
Age	20-35	100 (100.0)	0 (0.0)	0.440
	36-55	1050 (99.0)	11 (1.0)	
	56-75	803 (98.4)	13 (1.6)	
	N.D.	16 (100.0)	0 (0.0)	
Gender	F	1449 (98.9)	16 (1.1)	0.649
	M	477 (98.6)	7 (1.4)	
	N.D.	39 (100.0)		
Occupation	HCW	1375 (98.8)	16 (1.2)	0.751
	N-HCW	563 (98.6)	8 (1.4)	
	N.D.	29 (100.0)	0 (0.0)	
Seniority	1-5	107 (100.0)	0 (0.0)	0.639
	6-15	521 (98.7)	7 (1.3)	
	≥16	1317 (98.7)	17 (1.3)	
	N.D.	22 (100.0)	0 (0.0)	

Missing data analysis: Fear of Infection		Not missing	Missing	p
Age	20-35	100 (100.0)	0 (0.0)	0.692
	36-55	1052 (99.2)	9 (0.8)	
	56-75	807 (98.9)	9 (1.1)	
	N.D.	16 (100.0)	0 (0.0)	
Gender	F	1455 (99.3)	10 (0.7)	0.124
	M	476 (98.3)	8 (1.7)	
	N.D.	39 (100.0)		
Occupation	HCW	1376 (98.9)	15 (1.1)	0.438
	N-HCW	568 (99.5)	3 (0.5)	
	N.D.	29 (100.0)	0 (0.0)	
Seniority	1-5	107 (100.0)	0 (0.0)	0.683
	6-15	522 (98.9)	6 (1.1)	
	≥16	1322 (99.1)	12 (0.9)	
	N.D.	22 (100.0)	0 (0.0)	

Missing data analysis: Fear of Infecting Others		Not missing	Missing	p
Age	20-35	100 (100.0)	0 (0.0)	0.632
	36-55	1053 (99.2)	8 (0.8)	
	56-75	807 (98.9)	9 (1.1)	
	N.D.	16 (100.0)	0 (0.0)	
Gender	F	1457 (99.5)	8 (0.5)	0.052
	M	476 (98.3)	8 (1.7)	
	N.D.	39 (100.0)		
Occupation	HCW	1378 (99.1)	13 (0.9)	0.773
	N-HCW	567 (99.3)	4 (0.7)	
	N.D.	29 (100.0)	0 (0.0)	
Seniority	1-5	107 (100.0)	0 (0.0)	0.706

	6-15	524 (99.2)	4 (0.8)	
	≥16	1321 (99.0)	13 (1.0)	
	N.D.	22 (100.0)	0 (0.0)	

Missing data analysis: Obligation		Not missing	Missing	*p*
Age	20-35	100 (100.0)	0 (0.0)	0.632
	36-55	1053 (99.2)	8 (0.8)	
	56-75	807 (98.9)	9 (1.1)	
	N.D.	16 (100.0)	0 (0.0)	
Gender	F	1456 (99.4)	9 (0.6)	0.083
	M	476 (98.3)	8 (1.7)	
	N.D.	39 (100.0)		
Occupation	HCW	1379 (99.1)	12 (0.9)	0.830
	N-HCW	567 (99.3)	4 (0.7)	
	N.D.	29 (100.0)	0 (0.0)	
Seniority	1-5	107 (100.0)	0 (0.0)	0.753
	6-15	523 (99.1)	5 (0.9)	
	≥16	1322 (99.1)	12 (0.9)	
	N.D.	22 (100.0)	0 (0.0)	

Missing data analysis: Being Infected		Not missing	Missing	*p*
Age	20-35	100 (100.0)	0 (0.0)	0.632
	36-55	1053 (99.2)	8 (0.8)	
	56-75	807 (98.9)	9 (1.1)	
	N.D.	16 (100.0)	0 (0.0)	
Gender	F	1455 (99.3)	10 (0.7)	0.420
	M	478 (98.8)	6 (1.2)	
	N.D.	39 (100.0)		
Occupation	HCW	1376 (98.9)	15 (1.1)	0.438
	N-HCW	568 (99.5)	3 (0.5)	
	N.D.	29 (100.0)	0 (0.0)	
Seniority	1-5	107 (100.0)	0 (0.0)	0.750
	6-15	524 (99.2)	4 (0.8)	
	≥16	1322 (99.1)	12 (0.9)	
	N.D.	22 (100.0)	0 (0.0)	

Missing data analysis: Infecting Others		Not missing	Missing	*p*
Age	20-35	100 (100.0)	0 (0.0)	0.587
	36-55	1049 (98.9)	12 (1.1)	
	56-75	810 (99.3)	6 (0.7)	
	N.D.	16 (100.0)	0 (0.0)	
Gender	F	1451 (99.0)	14 (1.0)	0.661
	M	481 (99.4)	3 (0.6)	
	N.D.	39 (100.0)		
Occupation	HCW	1377 (99.0)	14 (1.0)	0.708

	N-HCW	567 (99.3)	4 (0.7)	
	N.D.	29 (100.0)	0 (0.0)	
Seniority	1-5	107 (100.0)	0 (0.0)	0.527
	6-15	525 (99.4)	3 (0.6)	
	≥16	1320 (99.0)	14 (1.0)	
	N.D.	22 (100.0)	0 (0.0)	

Missing data analysis: Efficacy		Not missing	Missing	*p*
Age	20-35	100 (100.0)	0 (0.0)	0.674
	36-55	1047 (98.7)	14 (1.3)	
	56-75	806 (98.8)	10 (1.2)	
	N.D.	16 (100.0)	0 (0.0)	
Gender	F	1449 (98.9)	16 (1.1)	0.772
	M	478 (98.8)	6 (1.2)	
	N.D.	39 (100.0)		
Occupation	HCW	1375 (98.8)	16 (1.2)	0.617
	N-HCW	562 (98.4)	9 (1.6)	
	N.D.	29 (100.0)	0 (0.0)	
Seniority	1-5	107 (100.0)	0 (0.0)	0.689
	6-15	522 (98.9)	6 (1.1)	
	≥16	1319 (98.9)	15 (1.1)	
	N.D.	22 (100.0)	0 (0.0)	

Missing data analysis: Safety		Not missing	Missing	*p*
Age	20-35	100 (100.0)	0 (0.0)	0.295
	36-55	1047 (98.7)	14 (1.3)	
	56-75	799 (97.9)	17 (2.1)	
	N.D.	16 (100.0)	0 (0.0)	
Gender	F	1444 (98.6)	21 (1.4)	0.454
	M	474 (97.9)	10 (2.1)	
	N.D.	39 (100.0)		
Occupation	HCW	1369 (98.4)	22 (1.6)	0.792
	N-HCW	562 (98.4)	9 (1.6)	
	N.D.	29 (100.0)	0 (0.0)	
Seniority	1-5	107 (100.0)	0 (0.0)	0.545
	6-15	521 (98.7)	7 (1.3)	
	≥16	1313 (98.4)	21 (1.6)	
	N.D.	22 (100.0)	0 (0.0)	

Missing data analysis: Effective		Not missing	Missing	*p*
Age	20-35	99 (99.0)	1 (1.0)	0.931
	36-55	1053 (99.2)	8 (0.8)	
	56-75	808 (99.0)	8 (1.0)	
	N.D.	16 (100.0)	0 (0.0)	
Gender	F	1456 (99.4)	9 (0.6)	0.332

	M	478 (98.8)	6 (1.2)	
	N.D.	39 (100.0)		
Occupation	HCW	1378 (99.1)	13 (0.9)	0.868
	N-HCW	566 (99.1)	5 (0.9)	
	N.D.	29 (100.0)	0 (0.0)	
Seniority	1-5	107 (100.0)	0 (0.0)	0.464
	6-15	525 (99.4)	3 (0.6)	
	≥16	1319 (98.9)	15 (1.1)	
	N.D.	22 (100.0)	0 (0.0)	

Missing data analysis: Safe		Not missing	Missing	*p*
Age	20-35	100 (100.0)	0 (0.0)	0.879
	36-55	1055 (99.4)	6 (0.6)	
	56-75	812 (99.5)	4 (0.5)	
	N.D.	16 (100.0)	0 (0.0)	
Gender	F	1458 (99.5)	7 (0.5)	0.840
	M	481 (99.4)	3 (0.6)	
	N.D.	39 (100.0)		
Occupation	HCW	1381 (99.3)	10 (0.7)	0.310
	N-HCW	570 (99.8)	1 (0.2)	
	N.D.	29 (100.0)	0 (0.0)	
Seniority	1-5	107 (100.0)	0 (0.0)	0.785
	6-15	526 (99.6)	2 (0.4)	
	≥16	1326 (99.4)	8 (0.6)	
	N.D.	22 (100.0)	0 (0.0)	

Missing data analysis: Updated		Not missing	Missing	*p*
Age	20-35	99 (99.0)	1 (1.0)	0.446
	36-55	1056 (99.5)	5 (0.5)	
	56-75	807 (98.9)	9 (1.1)	
	N.D.	16 (100.0)	0 (0.0)	
Gender	F	1454 (99.2)	11 (0.8)	0.831
	M	481 (99.4)	3 (0.6)	
	N.D.	39 (100.0)		
Occupation	HCW	1376 (98.9)	15 (1.1)	0.112
	N-HCW	570 (99.8)	1 (0.2)	
	N.D.	29 (100.0)	0 (0.0)	
Seniority	1-5	107 (100.0)	0 (0.0)	0.354
	6-15	526 (99.6)	2 (0.4)	
	≥16	1320 (99.0)	14 (1.0)	
	N.D.	22 (100.0)	0 (0.0)	

Missing data analysis: Booster Dose		Not missing	Missing	*p*
Age	20-35	99 (99.0)	1 (1.0)	0.978
	36-55	1049 (98.9)	12 (1.1)	

	56-75	807 (98.9)	9 (1.1)	
	N.D.	16 (100.0)	0 (0.0)	
Gender	F	1447 (98.8)	18 (1.2)	0.612
	M	480 (99.2)	4 (0.8)	
	N.D.	39 (100.0)		
Occupation	HCW	1377 (99.0)	14 (1.0)	0.779
	N-HCW	564 (98.8)	7 (1.2)	
	N.D.	29 (100.0)	0 (0.0)	
Seniority	1-5	106 (99.1)	1 (0.9)	0.820
	6-15	521 (98.7)	7 (1.3)	
	≥16	1322 (99.1)	12 (0.9)	
	N.D.	22 (100.0)	0 (0.0)	
